# p53-armed oncolytic adenovirus induces autophagy and apoptosis in KRAS and BRAF-mutant colorectal cancer cells

**DOI:** 10.1371/journal.pone.0294491

**Published:** 2023-11-16

**Authors:** Shuta Tamura, Hiroshi Tazawa, Naoto Hori, Yuncheng Li, Motohiko Yamada, Satoru Kikuchi, Shinji Kuroda, Yasuo Urata, Shunsuke Kagawa, Toshiyoshi Fujiwara

**Affiliations:** 1 Department of Gastroenterological Surgery, Okayama University Graduate School of Medicine, Dentistry and Pharmaceutical Sciences, Okayama, Japan; 2 Center for Innovative Clinical Medicine, Okayama University Hospital, Okayama, Japan; 3 Oncolys BioPharma, Inc., Tokyo, Japan; CNR, ITALY

## Abstract

Colorectal cancer (CRC) cells harboring KRAS or BRAF mutations show a more-malignant phenotype than cells with wild-type KRAS and BRAF. KRAS/BRAF–wild-type CRCs are sensitive to epidermal growth factor receptor (EGFR)-targeting agents, whereas KRAS/BRAF–mutant CRCs are resistant due to constitutive activation of the EGFR-downstream KRAS/BRAF signaling pathway. Novel therapeutic strategies to treat KRAS/BRAF mutant CRC cells are thus needed. We recently demonstrated that the telomerase-specific replication-competent oncolytic adenoviruses OBP-301 and p53-armed OBP-702 exhibit therapeutic potential against KRAS-mutant human pancreatic cancer cells. In this study, we evaluated the therapeutic potential of OBP-301 and OBP-702 against human CRC cells with differing KRAS/BRAF status. Human CRC cells with wild-type KRAS/BRAF (SW48, Colo320DM, CACO-2), mutant KRAS (DLD-1, SW620, HCT116), and mutant BRAF (RKO, HT29, COLO205) were used in this study. The antitumor effect of OBP-301 and OBP-702 against CRC cells was analyzed using the XTT assay. Virus-mediated modulation of apoptosis, autophagy, and the EGFR-MEK-ERK and AKT-mTOR signaling pathways was analyzed by Western blotting. Wild-type and KRAS-mutant CRC cells were sensitive to OBP-301 and OBP-702, whereas BRAF-mutant CRC cells were sensitive to OBP-702 but resistant to OBP-301. Western blot analysis demonstrated that OBP-301 induced autophagy and that OBP-702 induced autophagy and apoptosis in human CRC cells. In BRAF-mutant CRC cells, OBP-301 and OBP-702 suppressed the expression of EGFR, MEK, ERK, and AKT proteins, whereas mTOR expression was suppressed only by OBP-702. Our results suggest that p53-armed oncolytic virotherapy is a viable therapeutic option for treating KRAS/BRAF-mutant CRC cells via induction of autophagy and apoptosis.

## Introduction

Colorectal cancer (CRC) is the third leading cause of death worldwide [1]. CRCs harboring KRAS and BRAF mutations are often refractory to chemotherapy, resulting in a poorer prognosis than cases involving wild-type KRAS and BRAF due to tumor recurrence and metastasis [2]. KRAS/BRAF wild-type CRCs are sensitive to the epidermal growth factor receptor (EGFR)-targeting agents cetuximab and panitumumab [3]. However, KRAS/BRAF–mutant CRCs are highly resistant to EGFR-targeting therapy due to constitutive activation of the EGFR-downstream RAS-RAF-MEK-ERK signaling pathway [3]. Moreover, microsatellite stable (MSS) CRC reportedly exhibits greater resistance to immunotherapy compared with microsatellite instable (MSI) CRC [4]. Therefore, novel therapeutic strategies that will improve the clinical outcome in patients with KRAS/BRAF–mutant MSS CRC are needed.

KRAS–mutant CRCs harboring mutations at codon 12 or 13 are associated with worse prognosis [5]. BRAF–mutant CRCs frequently exhibit V600E mutation, leading to poor prognosis [6]. Constitutive activation of the RAS-RAF-MEK-ERK pathway plays a crucial role in the resistance to EGFR-targeting agents in KRAS/BRAF–mutant CRC cells [7]. Targeting the RAS-RAF-MEK-ERK pathway using BRAF, MEK, and ERK inhibitors improves the resistance to EGFR-targeting therapy in KRAS/BRAF-mutant CRC cells [8]. Dual inhibition of the RAS-RAF-MEK-ERK and EGFR-downstream PI3K-AKT-mTOR pathways using BRAF, MEK, ERK, and EGFR inhibitors has been suggested to be effective for treating KRAS/BRAF–mutant CRC cells [9]. Therefore, novel therapeutic strategies that suppress the RAS-RAF-MEK-ERK and EGFR-PI3K-AKT-mTOR pathways is needed for treating KRAS/BRAF–mutant CRCs.

Oncolytic virotherapy has recently emerged as a novel antitumor therapy against CRCs [10]. Oncolytic viruses induce tumor-specific death of malignant tumor cells via modulated viral replication [11]. Telomerase activity is higher in malignant tumor cells than normal cells [12]. To target malignant tumor cells with telomerase activity, we developed two types of telomerase-specific replication-competent oncolytic adenoviruses, OBP-301 [13] and OBP-702 armed with the wild-type *p53* tumor suppressor gene [14]. We previously demonstrated that OBP-301 induces autophagy-related death in human lung cancer cells by suppressing EGFR expression [15]. Moreover, we recently demonstrated that OBP-301 and p53-armed OBP-702 exhibit high antitumor efficacy against KRAS-mutant human pancreatic cancer cells via the KRAS-MEK-ERK signaling pathway [16]. Therefore, we hypothesized that OBP-301 and OBP-702 would be effective for use in eliminating KRAS/BRAF–mutant CRC cells.

In the present study, we investigated the therapeutic potential of the telomerase-specific replication-competent oncolytic adenoviruses OBP-301 and p53-armed OBP-702 for eliminating human CRC cells with differing KRAS/BRAF mutation status and microsatellite stability. The ability of OBP-301 and OBP-702 to induce apoptosis and autophagy and modulate the EGFR-MEK-ERK and AKT-mTOR signaling pathways was analyzed by Western blotting.

## Materials and methods

### Cell lines

The human CRC cell line Colo320DM was obtained from the Japanese Collection of Research Bioresources Cell Bank (Osaka, Japan). The human CRC cell lines SW48, SW620, DLD-1, HCT116, HT29, and RKO were obtained from the American Type Culture Collection (Manassas, VA, USA). The human CRC cell lines CACO-2 and COLO205 were obtained from the Riken BioResource Research Center (Tsukuba, Ibaraki, Japan). Colo320DM cells were maintained in Dulbecco’s modified Eagle’s medium. SW48, SW620, DLD-1, and COLO205 cells were maintained in RPMI-1640 medium. CACO-2 and RKO cells were maintained in Eagle’s Minimal Essential Medium. HCT116 and HT-29 cells were maintained in McCoy’s 5A medium. All media were supplemented with 10% fetal bovine serum (FBS), 100 U/ml penicillin, and 100 μg/ml streptomycin. Culture medium for CACO-2 cells were supplemented with 20% FBS and 1% non-essential amino acids solution. The cells were routinely maintained at 37°C in a humidified atmosphere with 5% CO_2_.

### Recombinant adenoviruses

**Fig 1:**
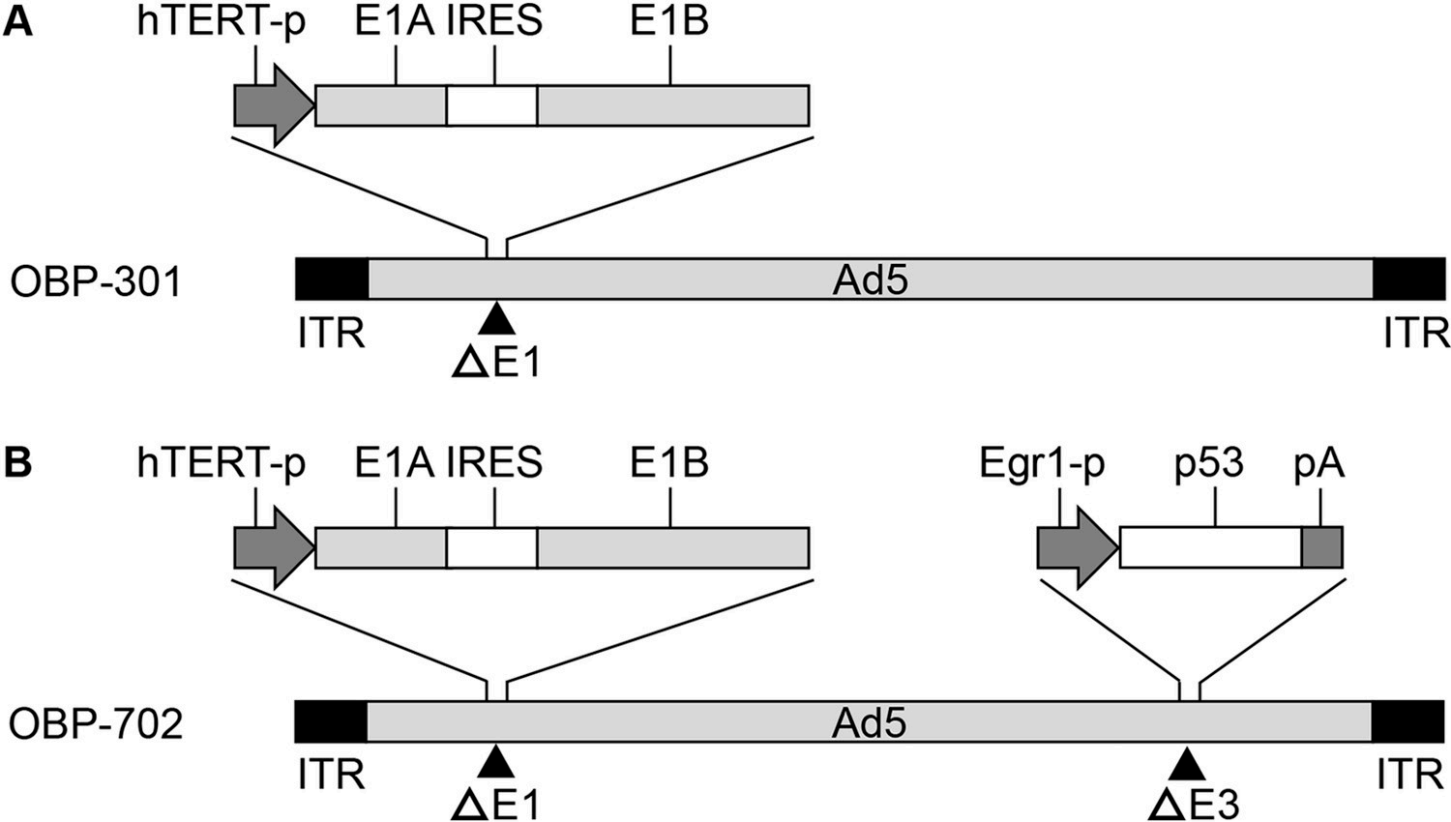
Structures of telomerase-specific replication-competent oncolytic adenoviruses. **A** OBP-301 is a telomerase-specific replication-competent oncolytic adenovirus in which the *hTERT* promoter drives expression of the *E1A* and *E1B* genes. **B** OBP-702 is a p53-armed OBP-301 variant in which the *Egr1* promoter drives expression of the human wild-type *p53* gene. Ad5, adenovirus serotype 5; hTERT, human telomerase reverse transcriptase; IRES, internal ribosome entry site; ITR, inverted terminal repeat.

The telomerase-specific replication-competent adenovirus OBP-301 (suratadenoturev), in which the promoter element of the human telomerase reverse transcriptase gene drives expression of the *E1A* and *E1B* genes, was previously constructed and characterized [13,17] (**Fig 1A**). OBP-702 was generated by modifying OBP-301 via insertion of a human wild-type *p53* gene expression cassette into the *E3* region of OBP-301 [14,18] (**Fig 1B**).

### Cell viability assay

Cells were seeded in 96-well plates at a density of 10^3^ cells/well. After 24 h, cells were infected with OBP-301 or OBP-702 at a multiplicity of infection (MOI) of 0, 1, 5, 10, 50, or 100 plaque-forming units (PFU)/cell. Uninfected (mock-treated) cells were used as virus-infected cells at an MOI of 0. Cell viability was determined on day 3 after virus infection using a Cell Proliferation Kit II (Roche Molecular Biochemicals, Indianapolis, IN, USA) according to the manufacturer’s protocol.

### Western blot analysis

Cells were seeded in a 100-mm dish at a density of 10^5^ cells/dish 24 h before virus infection. The cells were then infected for 72 h with OBP-301 or OBP-702 at the indicated MOI. Uninfected (mock-treated) cells were used as virus-infected cells at an MOI of 0. Whole-cell lysates were prepared in lysis buffer (50 mM Tris-HCl [pH 7.4], 150 mM NaCl, 1% Triton X-100) containing a protease inhibitor cocktail (Complete Mini; Roche Applied Science, Mannheim, Germany). Proteins (20 μg per lane) were electrophoresed on 6–10% SDS polyacrylamide gels and then transferred onto polyvinylidene difluoride membranes (Hybond-P; GE Health Care, Buckinghamshire, UK). Blots were blocked with Blocking One (Nacalai Tesque, Kyoto, Japan) at room temperature for 30 min. The primary antibodies used were as follows: rabbit anti–poly (ADP-ribose) polymerase (PARP) polyclonal antibody (pAb) (1:1000, 9542; Cell Signaling Technology, Danvers, MA, USA), rabbit anti-p62 pAb (1:1000, 5114; Cell Signaling Technology), mouse anti-E1A monoclonal antibody (mAb) (1:500, 554155; BD Bioscience, Franklin Lakes, NJ, USA), mouse anti-p53 mAb (1:1000, 18032; Cell Signaling Technology), rabbit anti-EGFR pAb (1:1000, 2232; Cell Signaling Technology), mouse anti-MEK1/2 mAb (1:1000, 4694; Cell Signaling Technology), rabbit anti-ERK1/2 mAb (1:1000, 4695; Cell Signaling Technology), rabbit anti-AKT mAb (1:1000, 4691; Cell Signaling Technology), rabbit anti-mTOR mAb (1:1000, 2983; Cell Signaling Technology), and mouse anti–β-Actin mAb (1:5000, A5441; Sigma-Aldrich, St. Louis, MO, USA). The secondary antibodies used were horseradish peroxidase–conjugated antibodies against mouse IgG (1:2500, NA931; GE Healthcare) or rabbit IgG (1:5000, NA934; GE Healthcare). Immunoreactive bands on the blots were visualized using enhanced chemiluminescence substrate (ECL Prime; GE Healthcare).

### Statistical analysis

Data are expressed as means ± SD. The significance of differences was assessed using the Student’s *t*-test. Statistical significance was defined as *P*<0.05.

## Results

### *In vitro* cytopathic effect of OBP-301 against human CRC cells with different KRAS/BRAF mutation status

**Fig 2:**
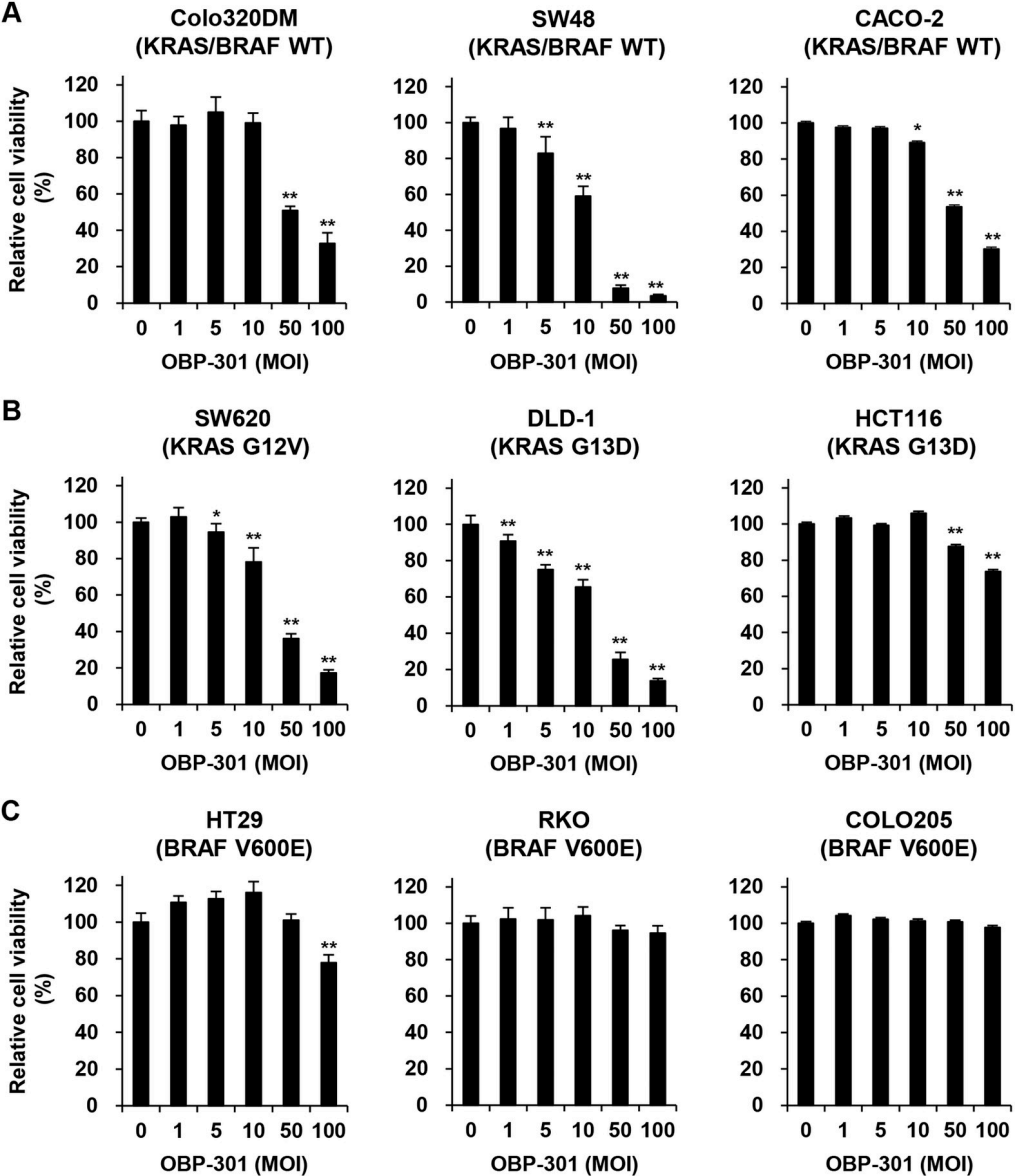
OBP-301 reduces the viability of human CRC cells with wild-type and mutant KRAS. **A, B, C** Human CRC cells with different KRAS/BRAF mutation status, KRAS/BRAF wild type (**A**), mutant KRAS (**B**), and mutant BRAF (**C**), were treated with OBP-301 at the indicated multiplicity of infection (MOI) for 72 h. Cell viability was quantified using the XTT assay. Uninfected (mock-treated) cells were shown as virus-infected cells at an MOI of 0. Cell viability was calculated relative to that of the mock-treated group, which was set at 100%. Cell viability data are expressed as mean ± SD (n = 5). The Student’s *t*-test was used to evaluate the significance of differences. *P<0.05; **P<0.01 (versus an MOI of 0).

To investigate the therapeutic potential of oncolytic adenoviruses against human CRC cells, we used three KRAS/BRAS wild-type human CRC cell lines, Colo320DM (MSS type), SW48 (MSI type), and CACO-2 (MSS type); three KRAS-mutant human CRC cell lines, SW620 (MSS type), DLD-1 (MSI type), and HCT116 (MSI type); and three BRAF-mutant human CRC cell lines, HT29 (MSS type), RKO (MSI type), and COLO205 (MSS type). The viability of CRC cells after infection with OBP-301 for 72 h was assessed using an XTT assay. OBP-301 treatment significantly suppressed the viability of KRAS/BRAF wild-type and KRAS-mutant CRC cells, independent of microsatellite status (**Fig 2A and 2B**). BRAF-mutant CRC cells were relatively less sensitive to OBP-301 compared with KRAS/BRAF wild-type or KRAS-mutant CRC cells (**Fig 2C**). These results suggest that OBP-301 has therapeutic potential against human CRC cells with wild-type KRAS/BRAF or mutant KRAS, but not mutant BRAF.

### OBP-301 induces autophagy in human CRC cells with different KRAS/BRAF mutation status

**Fig 3:**
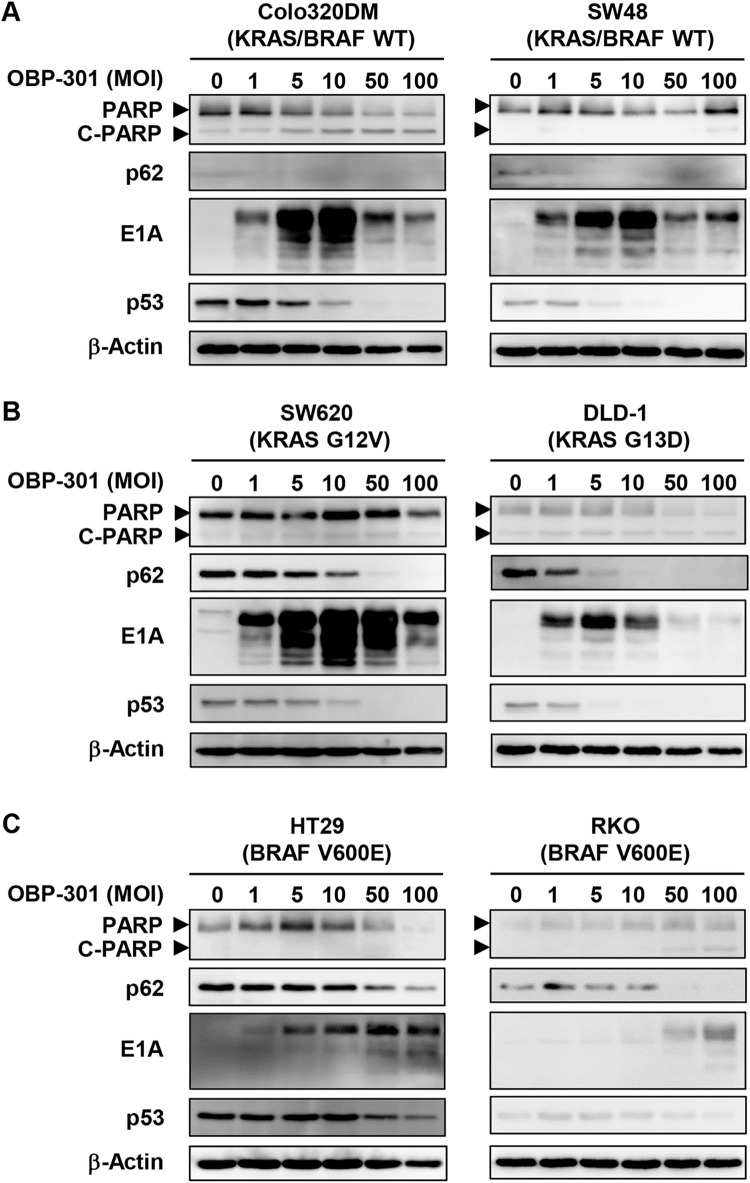
OBP-301 induces autophagy in human CRC cells. **A, B, C** Human CRC cells with different KRAS/BRAF mutation status, KRAS/BRAF wild type (**A**), mutant KRAS (**B**), and mutant BRAF (**C**), were treated with OBP-301 at the indicated MOI for 72 h. Cell lysates were prepared and subjected to Western blot analysis of PARP, cleaved PARP (C-PARP), p62, E1A, and p53 expression. β-Actin was assayed as a loading control. Uninfected (mock-infected) cells were shown as virus-infected cells at an MOI of 0.

Oncolytic adenoviruses have been shown to induce autophagy-related cell death in tumor cells [19–21]. We previously demonstrated that OBP-301 and OBP-702 exhibit cytopathic activity in association with autophagy and apoptosis in a variety of human cancer cells [15,18]. To investigate whether OBP-301 induces autophagy and apoptosis in human CRC cells, human CRC cells with different KRAS/BRAF mutation status were infected with OBP-301 for 72 h, and cell lysates were then prepared and subjected to Western blotting. OBP-301 treatment induced apoptosis with upregulation of C-PARP (but not autophagy due to a lack of p62 expression) in KRAS/BRAF wild-type Colo320DM cells (**Figs 3A and S1**). Following OBP-301 treatment, KRAS/BRAF wild-type SW48 cells exhibited autophagy with downregulation of p62 (**Figs 3A and S1**). OBP-301 treatment further induced autophagy (but not apoptosis) in KRAS-mutant and BRAF-mutant CRC cells (**Figs 3B, 3C, S2 and S3**). The expression of p53 protein was decreased by OBP-301 treatment in both p53-intact (SW48, RKO) and p53-mutant (Colo320DM, SW620, HT29, DLD-1) CRC cells (**Fig 3**). These results suggest that OBP-301 has therapeutic potential to induce autophagy in human CRC cells with wild-type KRAS/BRAF or mutant KRAS. However, OBP-301-induced autophagy was not effective to treat BRAF-mutant CRC cells.

### *In vitro* cytopathic effect of OBP-702 against human CRC cells with different KRAS/BRAF mutation status

**Fig 4:**
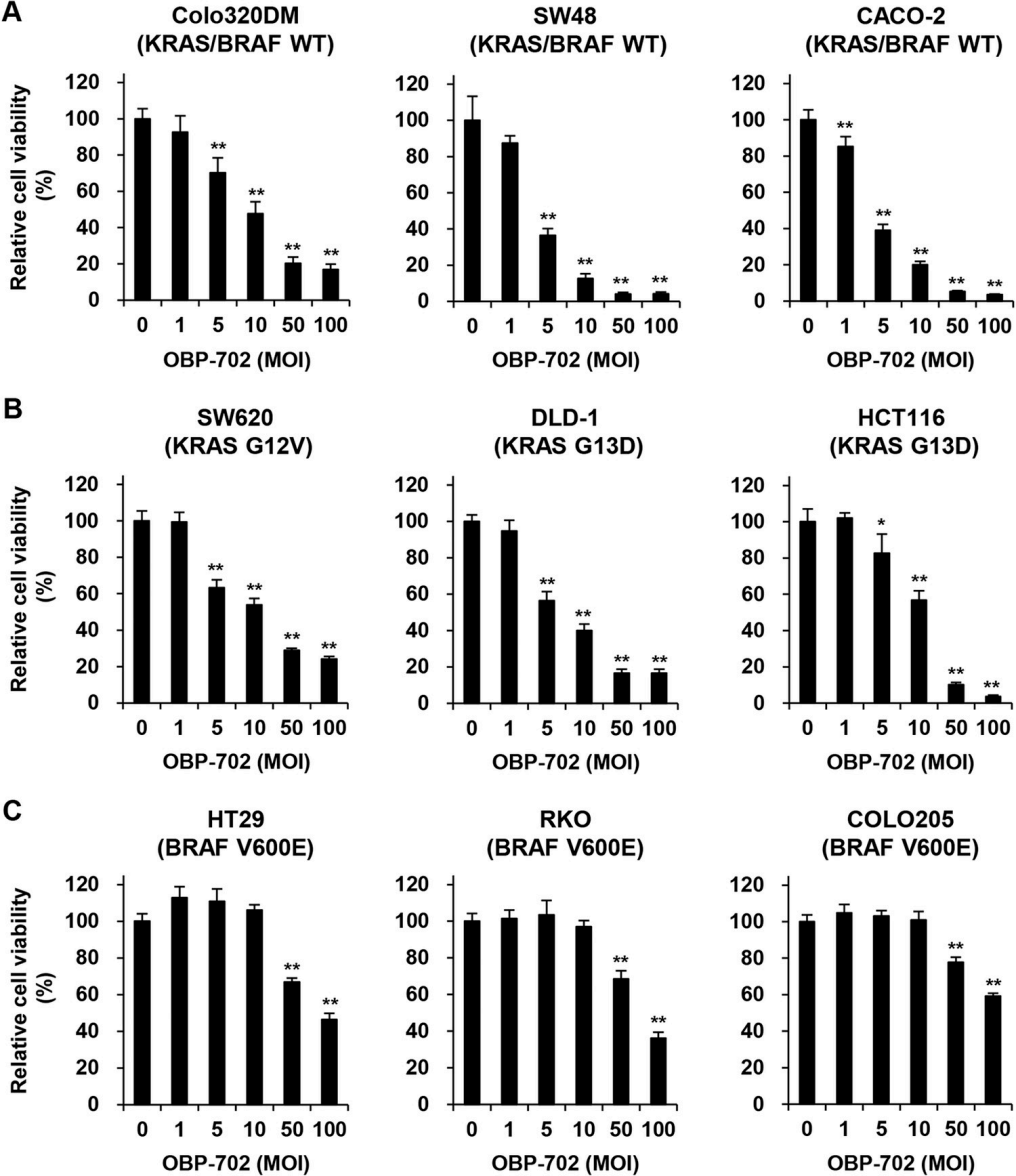
OBP-702 reduces the viability of human CRC cells independent of KRAS/BRAF mutation status. **A, B, C** Human CRC cells with different KRAS/BRAF mutation status, KRAS/BRAF wild type (**A**), mutant KRAS (**B**), and mutant BRAF (**C**), were treated with OBP-702 at the indicated MOI for 72 h. Cell viability was quantified using the XTT assay. Uninfected (mock-treated) cells were shown as virus-infected cells at an MOI of 0. Cell viability was calculated relative to that of the mock-treated group, which was set at 100%. Cell viability data are expressed as mean ± SD (n = 5). The Student’s *t*-test was used to evaluate the significance of differences. *P<0.05; **P<0.01 (versus an MOI of 0).

To investigate the therapeutic potential of p53-armed OBP-702 against human CRC cells, human CRC cells with different KRAS/BRAF mutation status were treated with OBP-702 for 72 h, and the viability of the cells was then assessed by XTT assay. OBP-702 treatment significantly decreased the viability of KRAS/BRAF wild-type, KRAS-mutant, and BRAF-mutant CRC cells, independent of microsatellite status (**Fig 4**). These results suggest that OBP-702 has therapeutic potential against human CRC cells independent of KRAS/BRAF mutation and microsatellite status.

### OBP-702 induces apoptosis and autophagy in human CRC cells with different KRAS/BRAF mutation status

**Fig 5:**
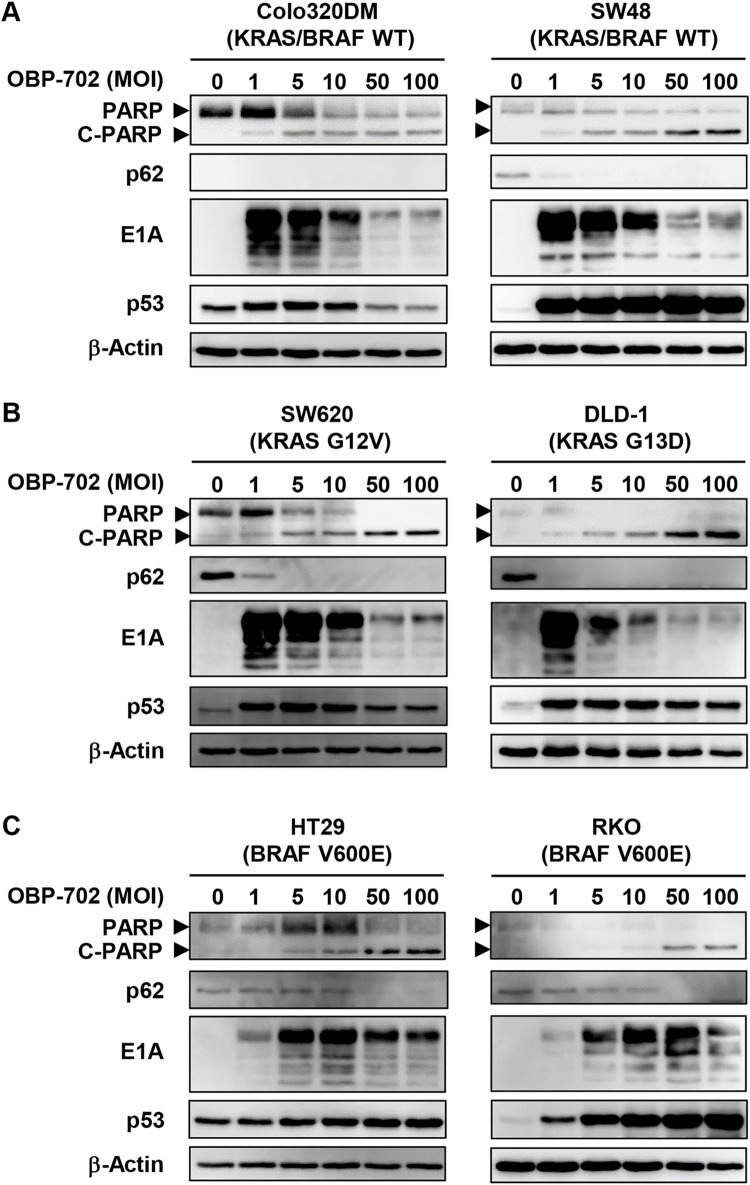
OBP-702 induces apoptosis and autophagy in human CRC cells. **A, B, C** Human CRC cells with different KRAS/BRAF mutation status, KRAS/BRAF wild type (**A**), mutant KRAS (**B**), and mutant BRAF (**C**), were treated with OBP-702 at the indicated MOI for 72 h. Cell lysates were prepared and subjected to Western blot analysis of PARP, cleaved PARP (C-PARP), p62, E1A, and p53 expression. β-Actin was assayed as a loading control. Uninfected (mock-infected) cells were shown as virus-infected cells at an MOI of 0.

To investigate whether OBP-702 induces autophagy and apoptosis in human CRC cells, human CRC cells with different KRAS/BRAF mutation status were infected with OBP-702 for 72 h, after which cell lysates were prepared and subjected to Western blotting. OBP-702 treatment induced apoptosis in all CRC cells with different KRAS/BRAF mutation status (**Figs 5 and S4–S6**). Moreover, OBP-702 treatment induced autophagy in all CRC cells except KRAS/BRAF wild-type Colo320DM cells, which lack p62 expression (**Figs 5 and S4–S6**). The expression of p53 protein was increased by OBP-702 treatment in both p53-intact (SW48, RKO) and p53-mutant (Colo320DM, SW620, HT29, DLD-1) CRC cells (**Figs 5 and S4–S6**). These results suggest that OBP-702 has therapeutic potential to induce apoptosis and autophagy in human CRC cells independent of KRAS/BRAF mutation status.

### OBP-702 suppresses the EGFR-MEK-ERK and AKT-mTOR signaling pathways in BRAF-mutant human CRC cells more strongly than OBP-301

**Fig 6:**
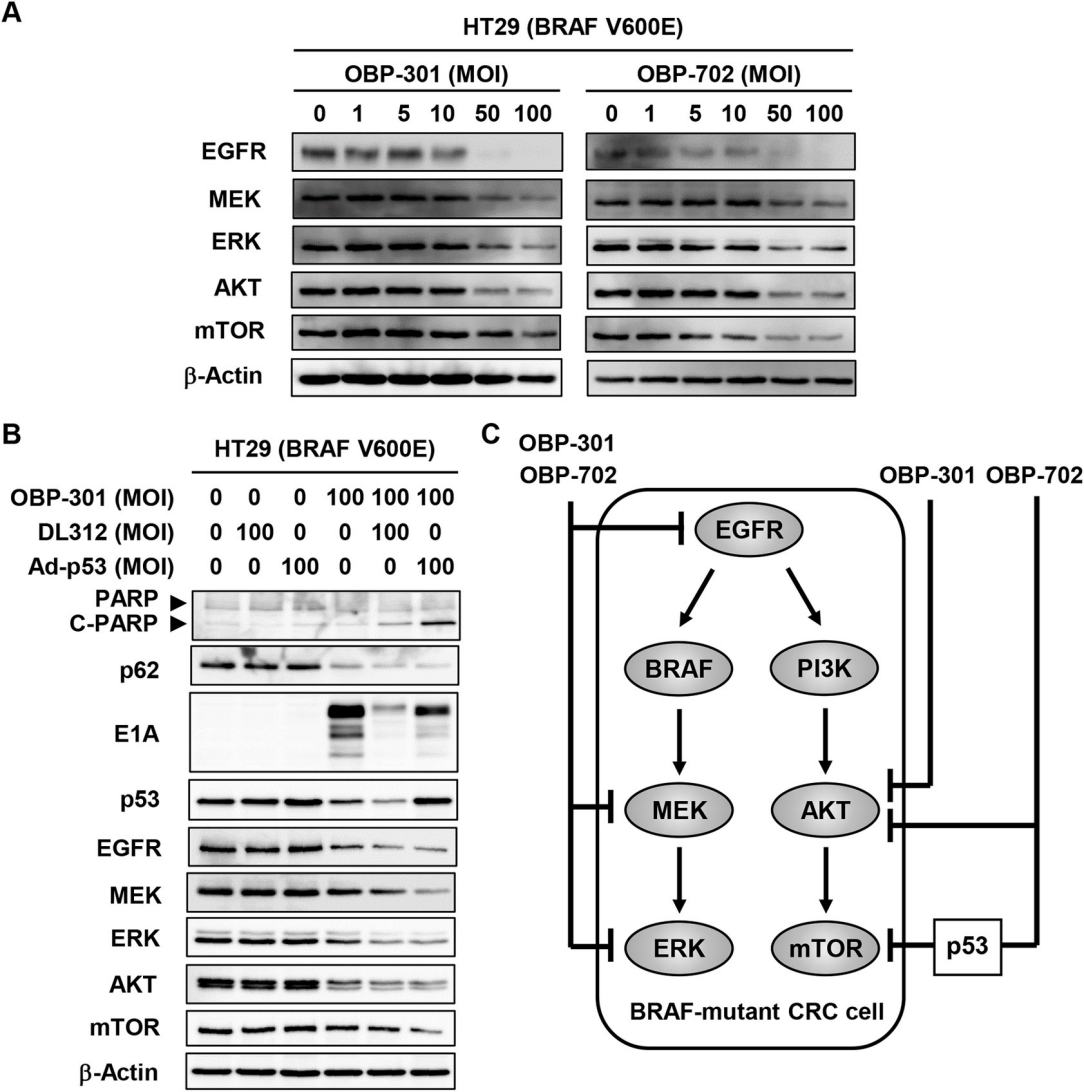
OBP-702 suppresses the EGFR-MEK-ERK and AKT-mTOR signaling pathways in BRAF-mutant CRC cells. **A** BRAF-mutant HT29 cells were treated with OBP-301 or OBP-702 at the indicated MOI for 72 h. Cell lysates were prepared and subjected to Western blot analysis of EGFR, MEK, ERK, AKT, and mTOR expression. **B** BRAF-mutant HT29 cells were treated with OBP-301, DL312, or Ad-p53 at the indicated MOI for 72 h. Cell lysates were prepared and subjected to Western blot analysis of PARP, C-PARP, p62, E1A, p53, EGFR, MEK, ERK, AKT, and mTOR expression. β-Actin was assayed as a loading control. Uninfected (mock-infected) cells were shown as virus-infected cells at an MOI of 0. **C** Outline of the EGFR-MEK-ERK and AKT-mTOR signaling pathways in BRAF-mutant CRC cells infected with OBP-301 or OBP-702.

BRAF-mutant CRC cells have been shown to exhibit activation of the EGFR-MEK-ERK and AKT-mTOR signaling pathways, resulting in malignant progression [22]. To explore the underlying mechanism of differing sensitivity to OBP-301 and OBP-702 in BRAF-mutant CRC cells, we investigated whether OBP-301 and OBP-702 suppress the EGFR-MEK-ERK and AKT-mTOR signaling pathways in BRAF-mutant CRC cells. BRAF-mutant HT29 cells were treated with OBP-301 or OBP-702 for 72 h, after which cell lysates were prepared and subjected to Western blotting. OBP-301 and OBP-702 efficiently suppressed the expression of EGFR, MEK, ERK, and AKT proteins (**Figs 6A, S7 and S8**). However, mTOR expression was suppressed by OBP-702 but not OBP-301 (**Figs 6A, S7 and S8**). These results suggest that OBP-702 is superior to OBP-301 in suppressing the AKT-mTOR signaling pathway, although the EGFR-MEK-ERK signaling pathway was similarly suppressed by OBP-301 and OBP-702.

To evaluate whether oncolytic virus–mediated p53 activation suppresses the expression of mTOR in BRAF-mutant CRC cells, BRAF-mutant HT29 cells were treated with OBP-301 and p53-expressing Ad-p53 or non-expressing control DL312 for 72 h. OBP-301 monotherapy induced autophagy with downregulation of p62, but it did not induce apoptosis, whereas treatment with Ad-p53 or DL312 did not induce either apoptosis or autophagy (**Figs 6B and S9**). Combination therapy with OBP-301 and Ad-p53 induced apoptosis with upregulation of C-PARP and p53, in addition to autophagy, more strongly than combination treatment with OBP-301 and DL312 (**Figs 6B and S9**). The expression of mTOR protein was suppressed by combination treatment with OBP-301 and Ad-p53 but not combination treatment with OBP-301 and DL312, although the expression of EGFR, MEK, ERK, and AKT proteins was similarly suppressed by combination treatment with OBP-301 and Ad-p53 or DL312 (**Figs 6B and S9**). These results suggest that oncolytic virus–mediated p53 activation plays a crucial role in suppression of the AKT-mTOR signaling pathway and apoptosis induction in BRAF-mutant CRC cells.

## Discussion

KRAS/BRAF-mutant CRC cells are thought to exhibit greater malignant potential in association with a greater likelihood of progression, metastasis, and resistance to EGFR-targeted therapies compared with KRAS/BRAF wild-type CRC cells [2,3]. Therefore, novel therapeutic strategies to treat KRAS/BRAF-mutant CRC are needed. In this study, we investigated the therapeutic potential of OBP-301 and OBP-702 against human CRC cells with different KRAS/BRAF mutation and microsatellite status. KRAS wild-type and KRAS-mutant CRC cells were sensitive to both OBP-301 and OBP-702 independent of microsatellite status. BRAF-mutant CRC cells were less sensitive to OBP-301 compared with OBP-702. OBP-301 induced autophagy in association with downregulation of p62 and p53 protein expression in CRC cells, whereas OBP-702 induced apoptosis and autophagy in association with upregulation of C-PARP and p53 protein expression in CRC cells. OBP-301 and OBP-702 efficiently suppressed the expression of EGFR, MEK, ERK, and AKT proteins in BRAF-mutant CRC cells (**Fig 6C**). However, mTOR expression was suppressed by OBP-702 but not OBP-301 (**Fig 6C**). Thus, p53-armed oncolytic virotherapy appears to be a promising antitumor strategy that strongly induces apoptosis and autophagy in KRAS/BRAF-mutant CRC cells by suppressing the EGFR-MEK-ERK and AKT-mTOR signaling pathways.

Oncolytic virotherapy with various viruses has been shown to induce the antitumor effect against KRAS/BRAF-mutant cancers, including CRC [23–26]. Jiffry et al. demonstrated that oncolytic reovirus-induced autophagy is effective to treat KRAS-mutant CRC cells [24]. Oncolytic adenovirus-induced autophagy has also been suggested to contribute to antitumor effect via mediating oncolysis and autophagy-related cell death [27]. We previously reported that OBP-301 induces autophagy-related cell death in human lung cancer cells by suppressing the EGFR expression [15]. OBP-301 and OBP-702 efficiently induced autophagy in KRAS/BRAF wild-type and KRAS-mutant CRC cells, although p53 expression was differentially modulated by OBP-301 and OBP-702. These findings suggest that autophagy induction may be involved in the therapeutic effect of OBP-301 and OBP-702 independent of p53 modulation. Autophagy plays both pro-survival and antitumoral roles in CRC cells [28]. The EGFR signaling pathway modulates autophagy in association with cell survival and cell death [29]. Li et al. demonstrated that the anti-EGFR antibody cetuximab induces autophagy in association with cell death in human CRC cells by activating the Beclin 1/hVps34 complex [30]. Giannopoulou et al. showed that the anti-EGFR antibody pantitumumab induces autophagy-related death in KRAS-mutant CRC cells by increasing the Beclin 1 protein level [31]. We also demonstrated that OBP-301 and OBP-702 induce autophagy-related death in KRAS-mutant human pancreatic cancer cells by suppressing the KRAS signaling pathway [16]. Thus, oncolytic virotherapy appears to be a promising antitumor strategy for inducing autophagy-related death in KRAS/BRAF wild-type and KRAS-mutant CRC cells by suppressing the EGFR-KRAS signaling pathway.

p53-armed OBP-702 induced apoptosis in BRAF-mutant CRC cells, whereas non-armed OBP-301 or p53-expressing Ad-p53 did not induce apoptosis, suggesting that p53-activating oncolytic virotherapy would be effective for treating BRAF-mutant CRC via apoptosis induction. With regard to the molecular mechanism of the OBP-702–mediated cytopathic effect against BRAF-mutant CRC cells, our data showed that OBP-702 suppresses the expression of mTOR protein in BRAF-mutant CRC cells. Accumulating evidence indicates that p53 activation inhibits the mTOR pathway at transcriptional and non-transcriptional levels [32]. With regard to the role of mTOR inhibition in BRAF-mutant CRC cells, Mao et al. demonstrated that suppression of the AKT-mTOR signaling pathway enhances the sensitivity to BRAF inhibition in BRAF-mutant CRC cells [33]. Garcia-Garcia et al. showed that dual blockade of the MEK-ERK and AKT-mTOR signaling pathways results in the induction of apoptosis in BRAF-mutant RKO cells (p53 wild-type) but not BRAF-mutant HT-29 cells (p53 mutant) [34], suggesting that p53 activation plays an important role in suppression of the MEK-ERK and AKT-mTOR signaling pathways, followed by apoptosis induction. Although whether suppression of mTOR is associated with OBP-702-induced apoptosis remains unclear, He et al. demonstrated that mTOR inhibitors induce apoptosis in KRAS/BRAF-mutant CRC cells via the extrinsic apoptotic pathway [35]. Thus, OBP-702 treatment may contribute to the induction of apoptosis in BRAF-mutant CRC cells by suppressing the AKT-mTOR and EGFR-MEK-ERK signaling pathways and activating the p53 signaling pathway.

Combination of oncolytic virotherapy with small molecules targeting the MEK-ERK pathway has also been shown to induce more profound antitumor efficacy than monotherapy in *in vivo* tumor models with KRAS/BRAF mutations [36]. Lee et al. showed that combination of MEK inhibitor promotes the antitumor efficacy of oncolytic vaccinia virus against chemo-resistant human ovarian cancer cells via enhancement of virus replication [37]. Bommareddy et al. demonstrated that combination of MEK inhibitor enhances antitumor effect of oncolytic herpes simplex virus against KRAS-mutant murine CRC tumors via enhancement of cytopathic activity and antitumor immunity [38]. Thus, *in vivo* experiments using immune-deficient and immune-competent mice are warranted to evaluate the therapeutic potential of OBP-301 and OBP-702 against KRAS/BRAF-mutant CRC tumors in monotherapy and combination therapy with small molecules targeting the MEK-ERK pathway.

Clinical application of OBP-301 and OBP-702 is expected as treatment modalities for CRC. Administration route of OBP-301 and OBP-702 is limited to intratumoral injection to avoid the antiviral immune response mediated by circulating neutralizing antibody. Therefore, rectal cancer may be suitable in treating accessible CRC tumors. We previously reported that intratumoral injection of OBP-301 suppresses lymph node metastasis in an orthotopic rectal tumor model with KRAS-mutant HCT116 cells [39]. It has also been shown that intratumoral injection of OBP-702 exhibits antitumor effect in subcutaneous and orthotopic tumor models with human pancreatic cancer cells harboring in-frame *BRAF* deletion [16]. More recently, we demonstrated that endoscopic intratumoral injection of OBP-301 with radiotherapy was feasible and well tolerated in esophageal cancer patients [40]. Although whether the feasibility and safety of intratumoral injection of OBP-702 in CRC patients remains to be elucidated, rectal cancers with KRAS/BRAF mutations may be potent candidate for treating with OBP-301 and OBP-702.

In conclusion, we demonstrated that the telomerase-specific oncolytic adenoviruses OBP-301 and OBP-702 have therapeutic potential for inducing autophagy-related death in KRAS wild-type and KRAS-mutant CRC cells. Moreover, OBP-702–mediated p53 activation may provide a novel therapeutic option for inducing apoptosis in BRAF-mutant CRC cells. *In vivo* experiments are needed to evaluate the therapeutic potential of OBP-301 and OBP-702 against KRAS/BRAF-mutant CRC tumors.

## Supporting information

S1 Fig Full image of Fig 3A. (PDF)

S2 Fig Full image of Fig 3B. (PDF)

S3 Fig Full image of Fig 3C. (PDF)

S4 Fig Full image of Fig 5A. (PDF)

S5 Fig Full image of Fig 5B. (PDF)

S6 Fig Full image of Fig 5C. (PDF)

S7 Fig Full image of Fig 6A (OBP-301). (PDF)

S8 Fig Full image of Fig 6A (OBP-702). (PDF)

S9 Fig Full image of Fig 6B. (PDF)

## Abbreviations

CRC: colorectal cancer
EGFR: epidermal growth factor receptor
mAb: monoclonal antibody
MOI: multiplicity of infection
MSI: microsatellite instable
MSS: microsatellite stable
pAb: polyclonal antibody
PARP: poly (ADP-ribose) polymerase
PFU: plaque-forming units
