# Supplementary figures and images for: p53-armed oncolytic adenovirus induces autophagy and apoptosis in KRAS and BRAF-mutant colorectal cancer cells
Source: PLoS One. 2023 Nov 16;18(11):e0294491. doi: 10.1371/journal.pone.0294491 (PMC10653454; doi:10.1371/journal.pone.0294491)

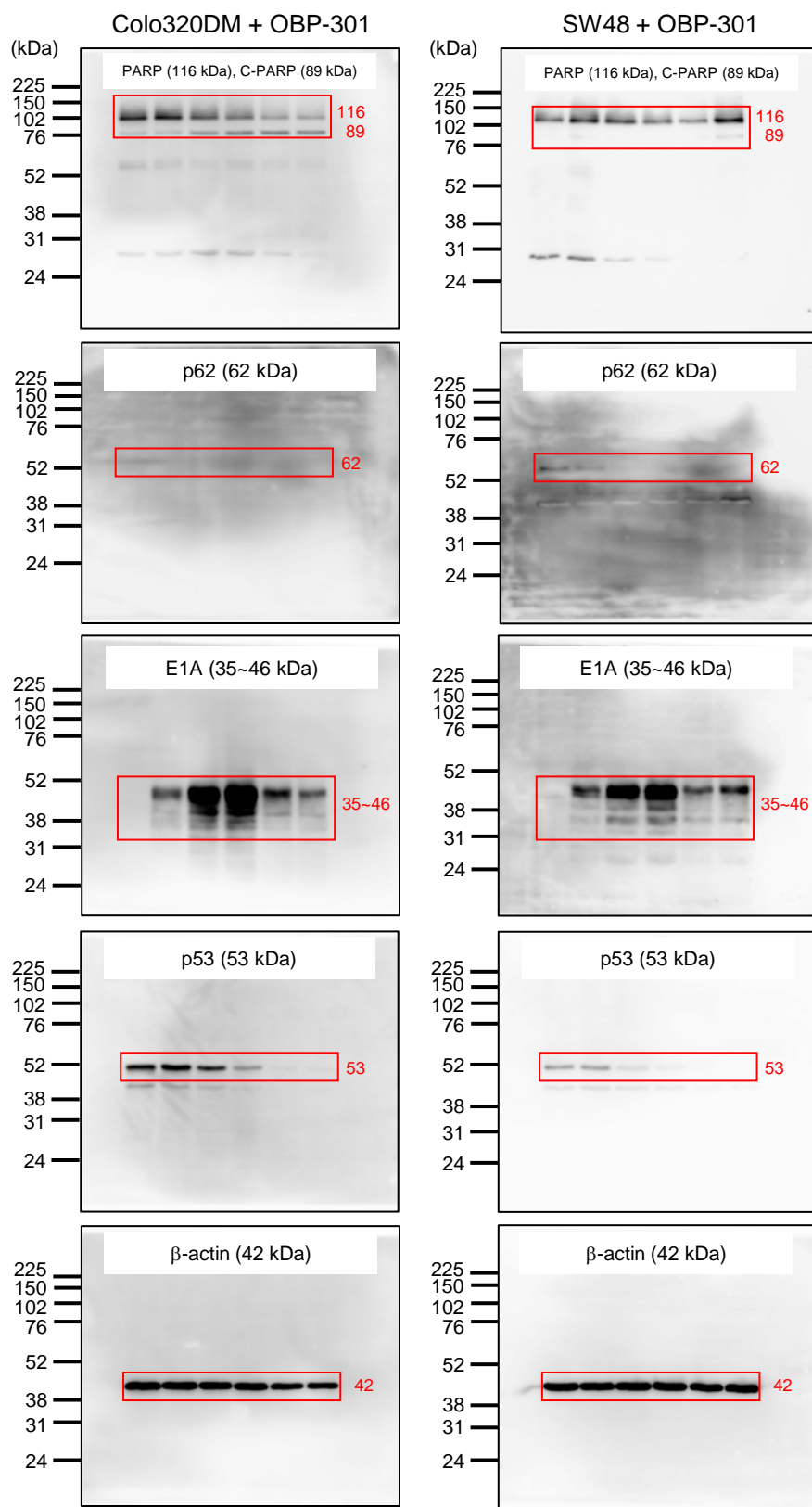

**S1 Figure**

**Full image of Figure 3A**

Supplement: S1 Fig — (PDF) [file pone.0294491.s001.pdf]

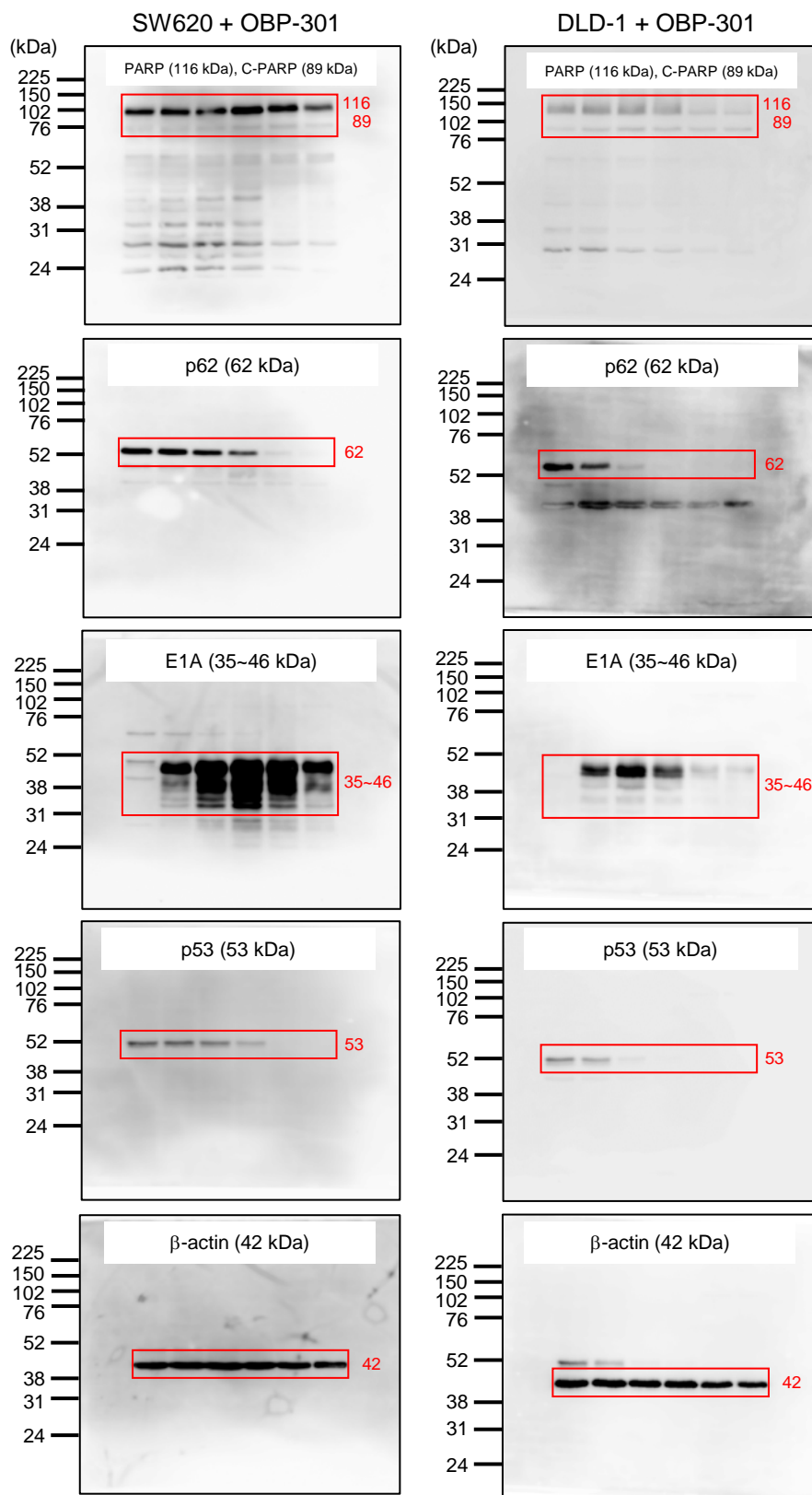

**S2 Figure**

**Full image of Figure 3B**

Supplement: S2 Fig — (PDF) [file pone.0294491.s002.pdf]

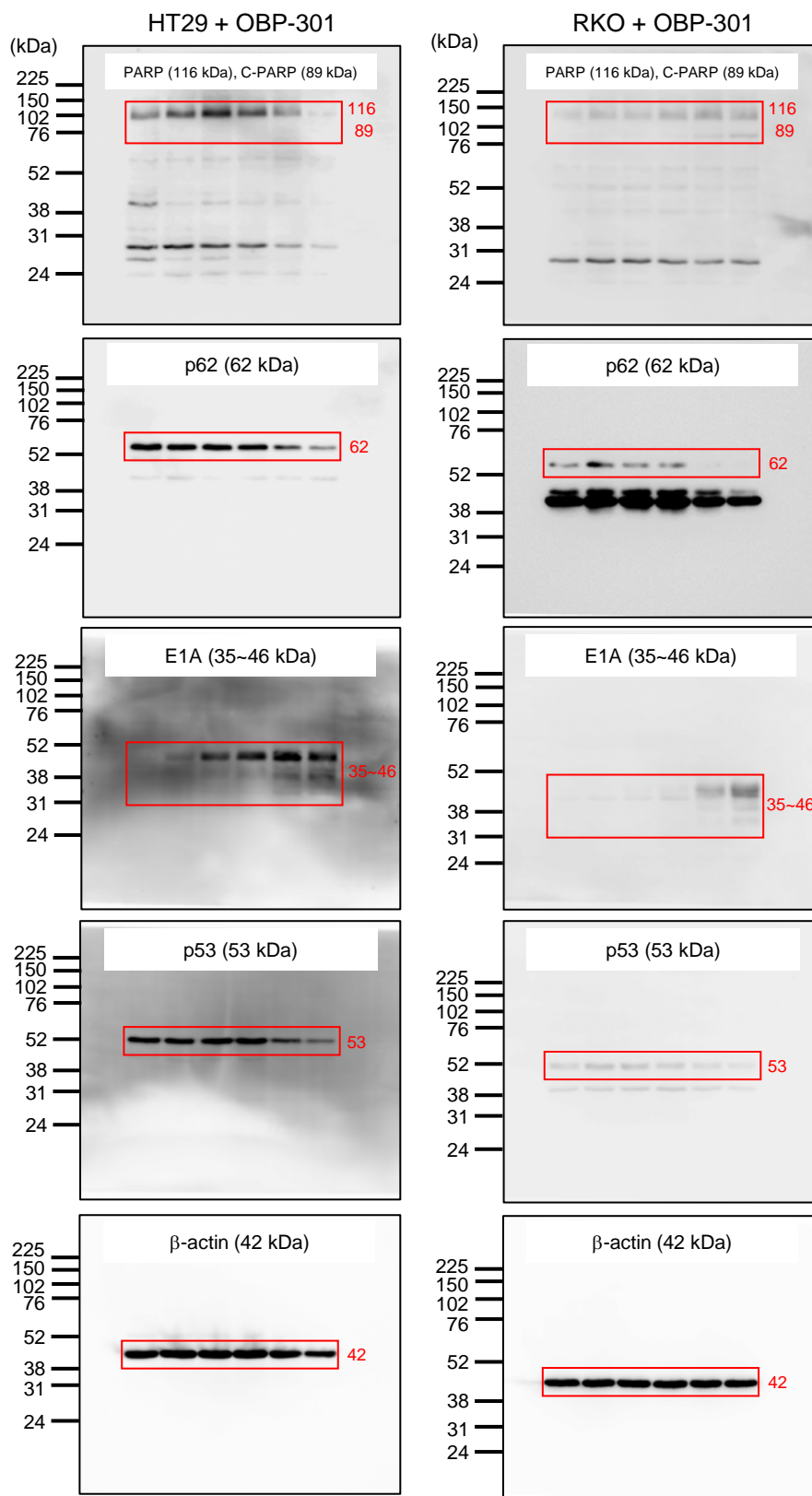

**S3 Figure**

**Full image of Figure 3C**

Supplement: S3 Fig — (PDF) [file pone.0294491.s003.pdf]

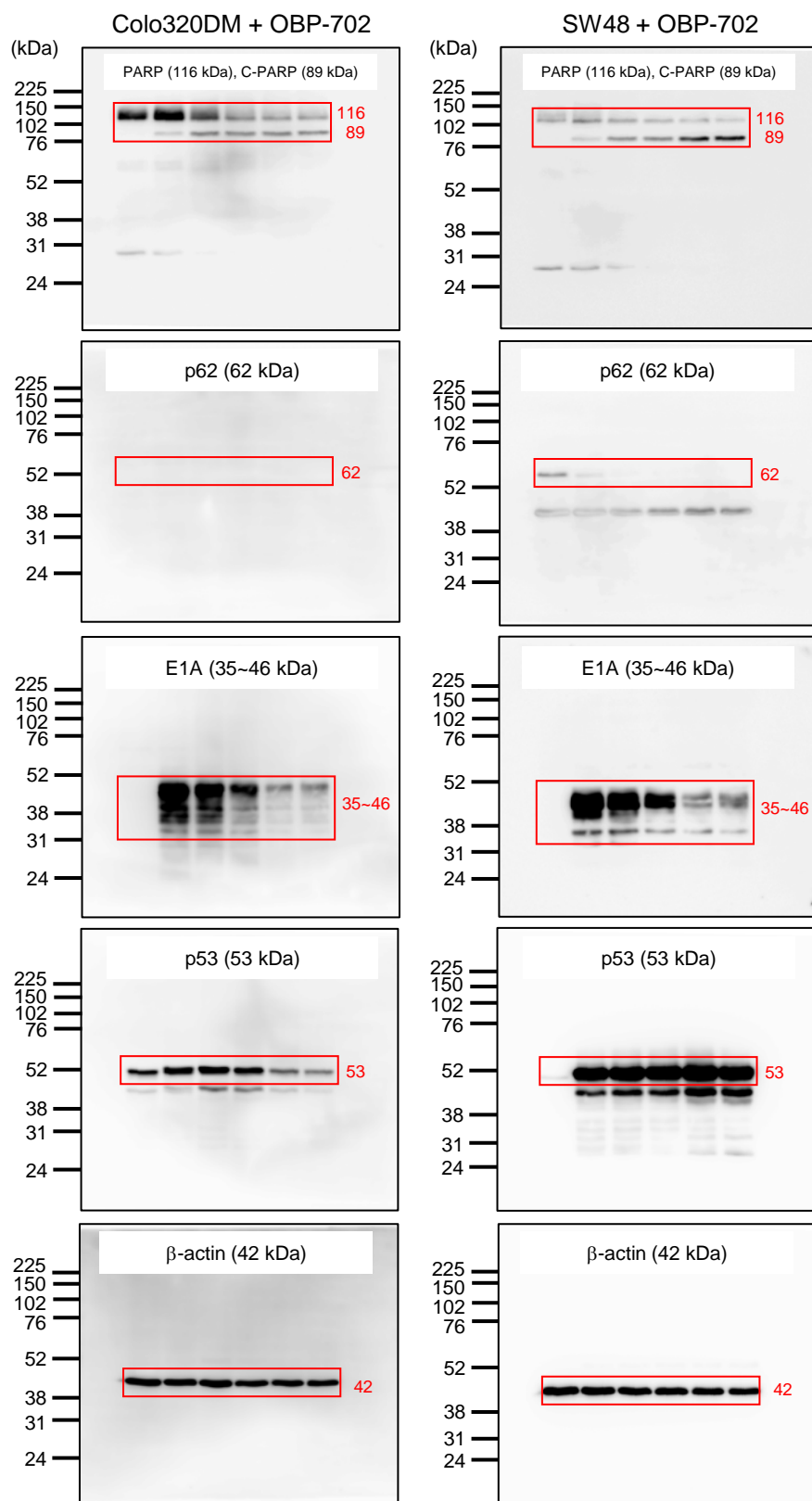

**S4 Figure**

**Full image of Figure 5A**

Supplement: S4 Fig — (PDF) [file pone.0294491.s004.pdf]

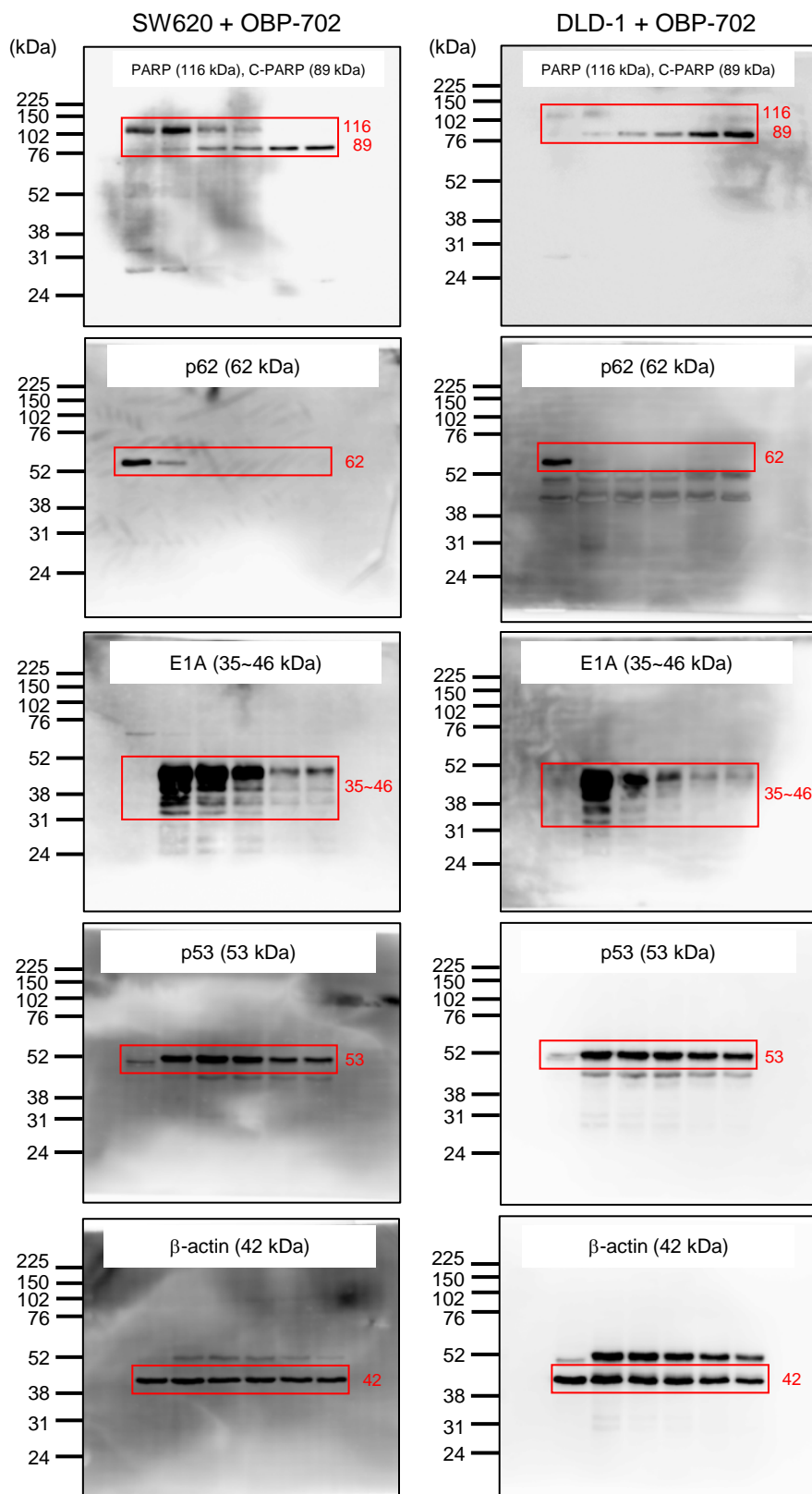

**S5 Figure**

Supplement: S5 Fig — (PDF) [file pone.0294491.s005.pdf]

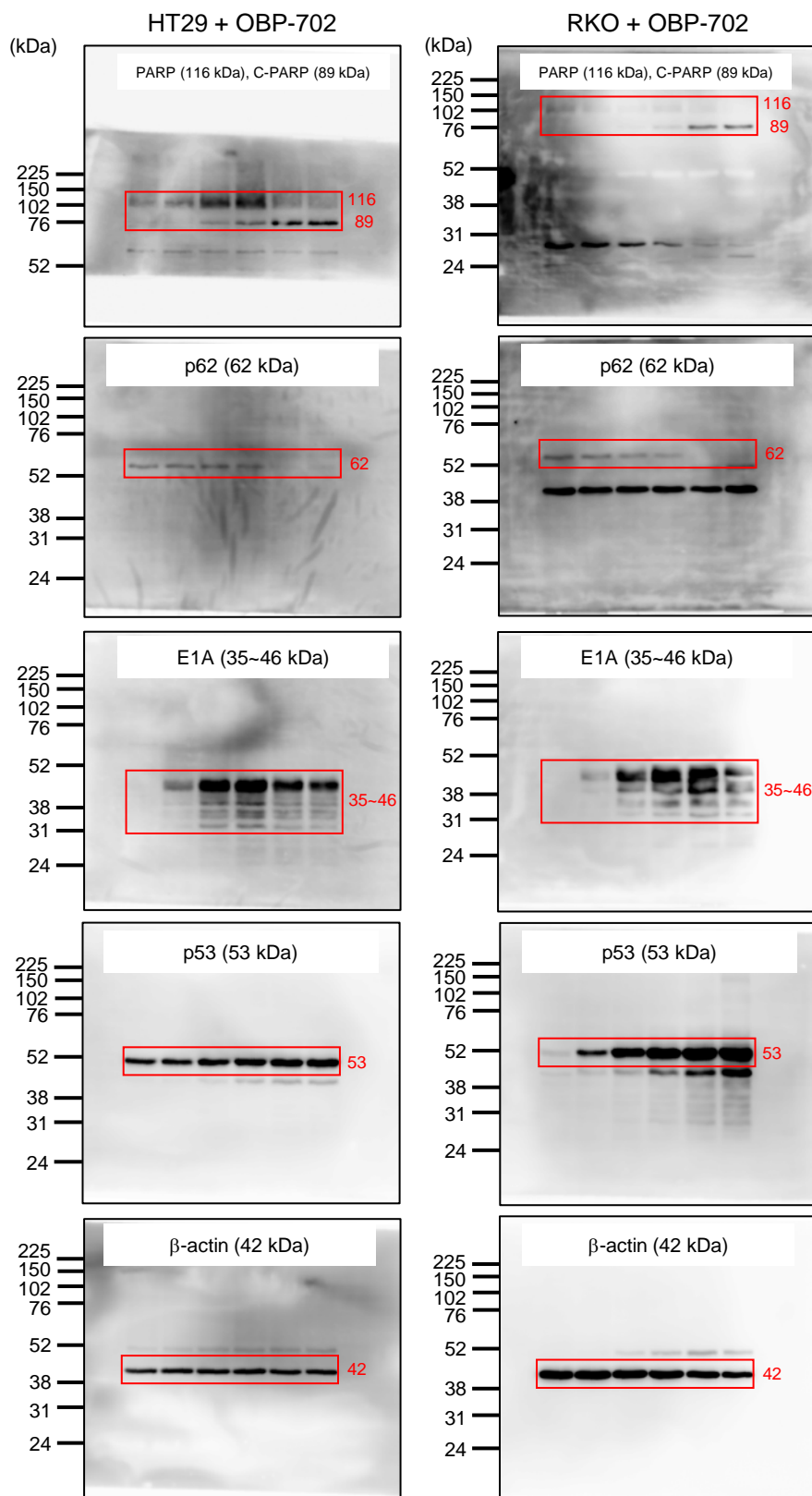

**S6 Figure**

Supplement: S6 Fig — (PDF) [file pone.0294491.s006.pdf]

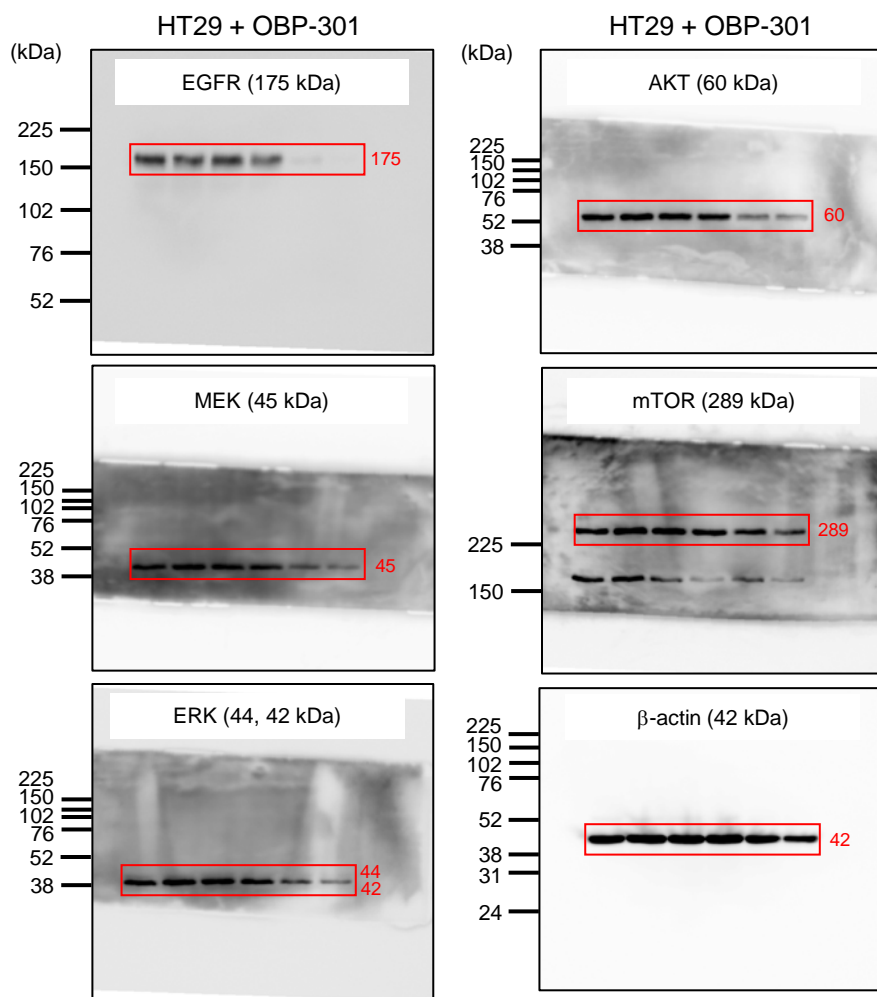

**S7 Figure**

**Full image of Figure 6A (OBP-301)**

Supplement: S7 Fig — (PDF) [file pone.0294491.s007.pdf]

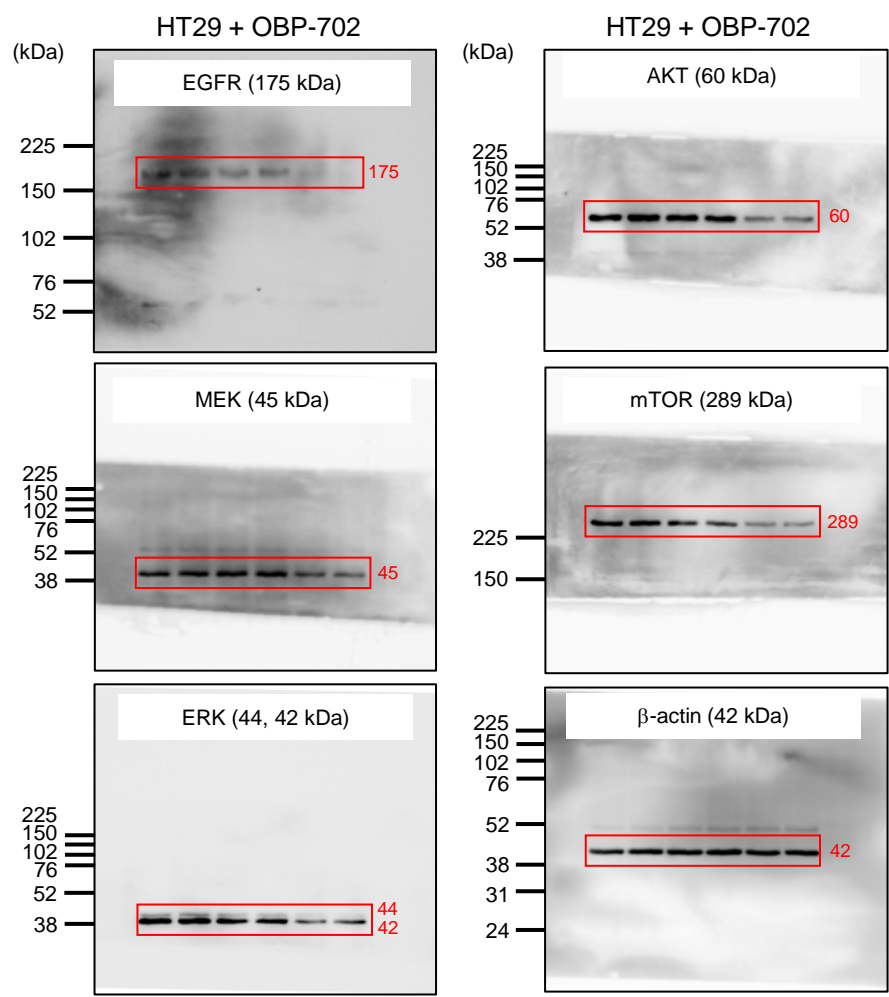

**S8 Figure**

**Full image of Figure 6A (OBP-702)**

Supplement: S8 Fig — (PDF) [file pone.0294491.s008.pdf]

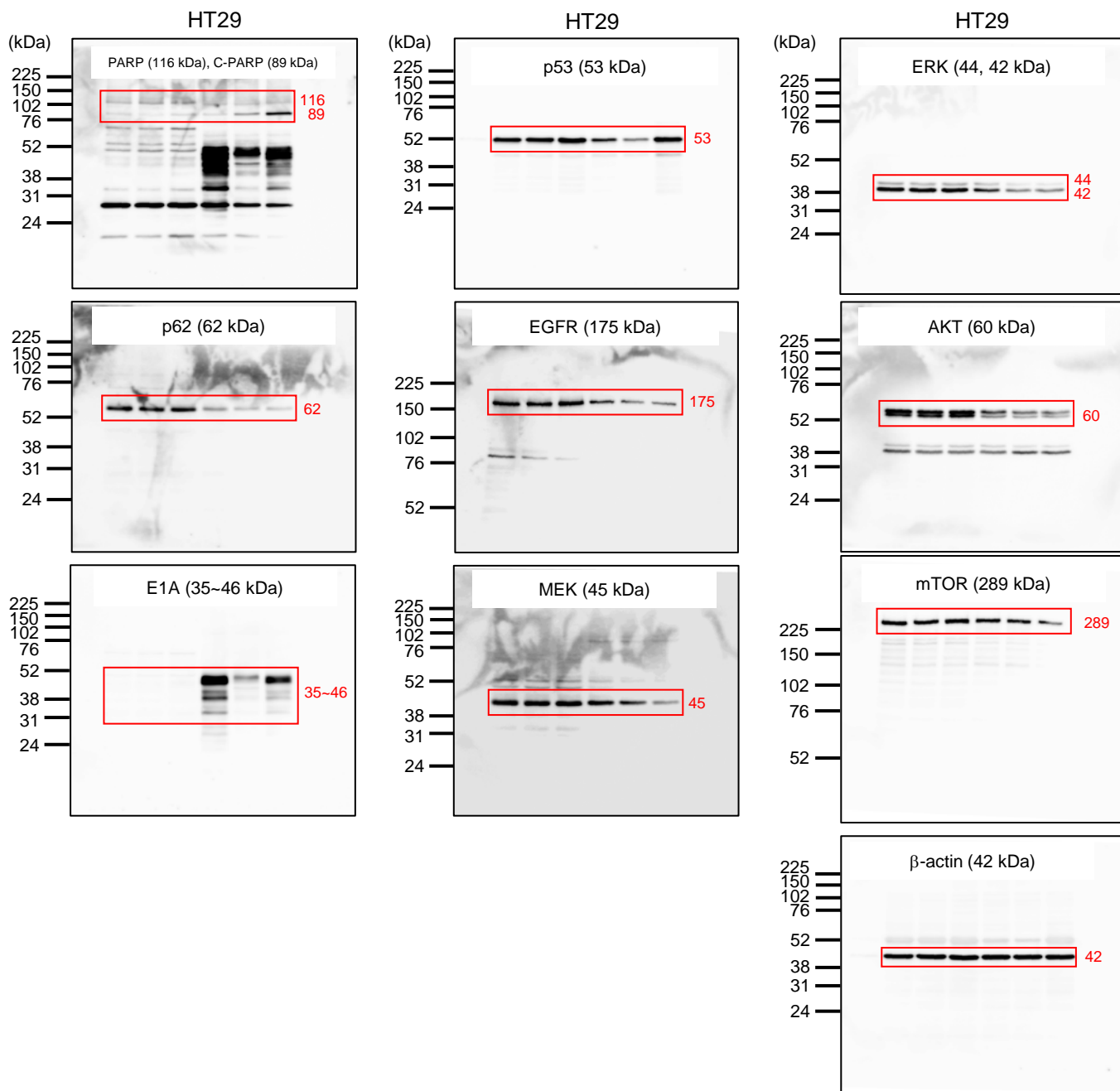

**S9 Figure**

**Full image of Figure 6B**

Supplement: S9 Fig — (PDF) [file pone.0294491.s009.pdf]
